# Supplementary material for: SIRT1 mediates obesity- and nutrient-dependent perturbation of pubertal timing by epigenetically controlling Kiss1 expression
Source: Nat Commun. 2018 Oct 10;9:4194. doi: 10.1038/s41467-018-06459-9 (PMC6179991; doi:10.1038/s41467-018-06459-9)
Supplement: Supplementary file 1 — Supplementary Information [file 41467_2018_6459_MOESM1_ESM.pdf]

SUPPLEMENTARY INFORMATION FILES

SIRT1 mediates obesity- and nutrient-dependent perturbation of pubertal timing by epigenetically controlling *Kiss1* expression

M.J. Vazquez[1,2,3,4]*, C.A. Toro[5]*, J.M. Castellano[1,2,3,4], F. Ruiz-Pino[1,2,3,4], J. Roa[1,2,3,4], D. Beiroa[6], V. Heras[1,2,3], I. Velasco[1,2,3,4], C. Dieguez[4,6], L. Pinilla[1,2,3,4], F. Gaytan[1,2], R. Nogueiras[4,6], M. A. Bosch[7], O.K. Rønnekleiv[7,8], A. Lomniczi[5], S.R. Ojeda[8], M. Tena-Sempere[1,2,3,4,9]

[1]Instituto Maimónides de Investigación Biomédica de Cordoba (IMIBIC); [2]Department of Cell Biology, Physiology and Immunology, University of Cordoba; [3]Hospital Universitario Reina Sofia; and [4]CIBER Fisiopatología de la Obesidad y Nutrición, Instituto de Salud Carlos III, 14004 Cordoba, Spain; [5]Division of Genetics, Oregon National Primate Research Center/Oregon Health and Science University, Beaverton, Oregon USA; [6]Department of Physiology, Faculty of Medicine and CIMUS, University of Santiago de Compostela-Instituto de Investigación Sanitaria, 15782 Santiago de Compostela, Spain; [7]Department of Physiology and Pharmacology, Oregon Health and Science University, Portland, Oregon, USA; [8]Division of Neuroscience, Oregon National Primate Research Center/Oregon Health and Science University, Beaverton, Oregon USA; and [9]FiDiPro Program, University of Turku, Turku, Finland

* These authors contributed equally to this work and should be considered joint first authors

Short Title: Obesity alters puberty onset via SIRT1/Kiss1

Key Words: Sirt1, sirtuins, Kiss1, kisspeptins, obesity, undernutrition, puberty

Corresponding authors: Alejandro Lomniczi (lomniczi@ohsu.edu) and
Manuel Tena-Sempere (fi1tesem@uco.es)
Department of Cell Biology, Physiology & Immunology
Faculty of Medicine, University of Córdoba
Avda. Menéndez Pidal s/n. 14004 Córdoba, SPAIN

SUPPLEMENTARY FIGURES

Supplementary Figure 1

Supplementary Figure 2

Supplementary Figure 3

Supplementary Figure 4

Supplementary Figure 5

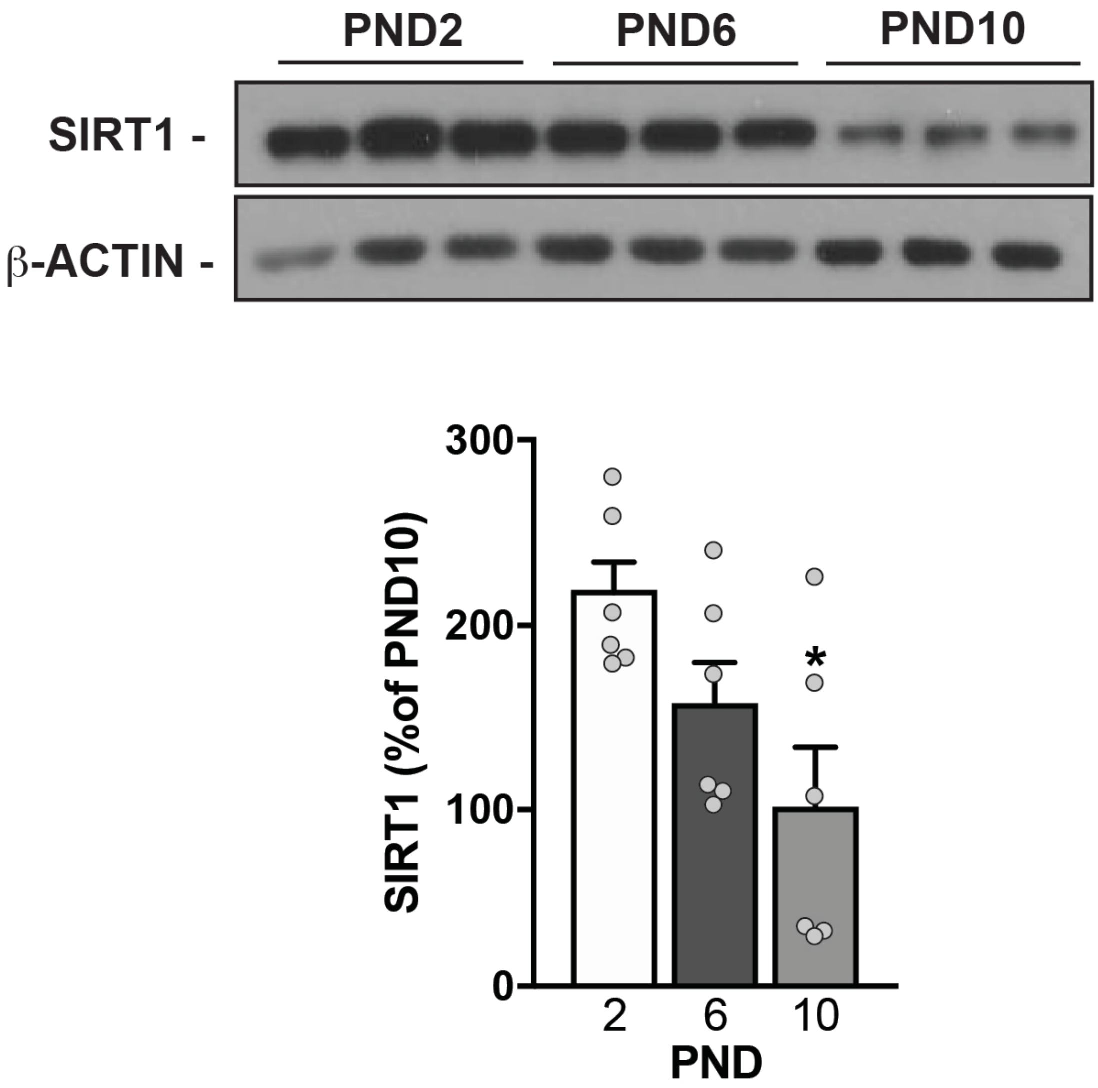

Supplementary Figure 1: SIRT1 content in the preoptic area (POA)-medial basal hypothalamus (MBH) unit of immature female rats, at postnatal day (PND) 2, 6 and 10. Representative Western blots are presented in the upper panel. The bar histograms represent the mean ± SEM. *$p<0.05$ (One way ANOVA followed by the Student-Newman-Keuls test). N=6 animals/group. PND2= white bar; PND6= dark grey bar; PND10= light grey bar.

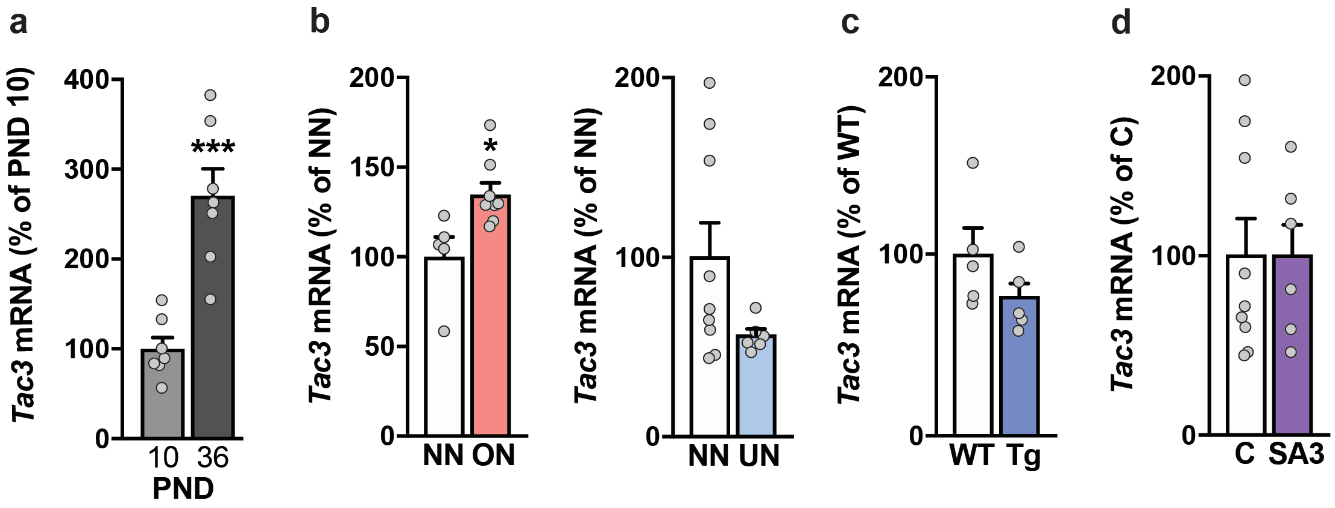

Supplementary Figure 2: Levels of *Tac3* mRNA in hypothalamic samples from various female rat models, with normal or altered puberty. (**a**) *Tac3* mRNA levels in the medial basal hypothalamus (MBH) of female rats, at postnatal day (PND) 10 (infantile) or PND36 (pubertal). (**b**) *Tac3*mRNA levels in the MBH of female rats, fed normally (NN) or subjected to postnatal overnutrition (ON; samples taken at PND29) or under-nutrition (UN; samples taken at PND36). (**c**) *Tac3* mRNA levels in the hypothalamus of a transgenic model of moderate *Sirt1* over-expression (Tg), and their respective WT controls, at PND32. (**d**) Hypothalamic *Tac3* mRNA levels in a rat model of central activation of SIRT1 with the allosteric activator, SA3. The bar histograms represent the mean ± SEM. *p<0.05; ***p<0.001 (two-sided Student-t test). PND10= light grey bars; PND36= dark grey bars; NN= white bars; ON= light red bars; UN= light blue bars; WT= white bars; Tg= cyan bars; SA3= violet bars.

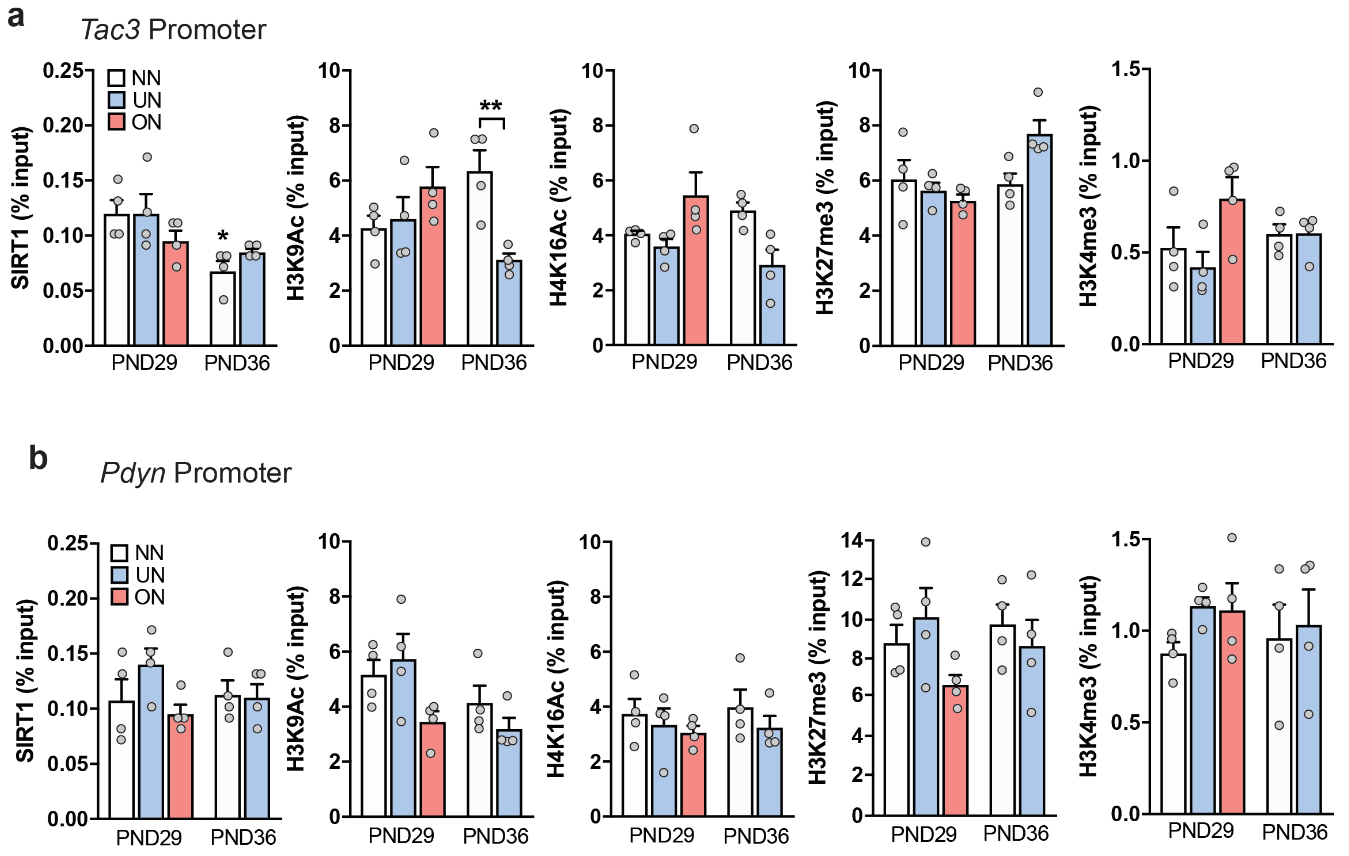

Supplementary Figure 3: Chromatin Immunoprecipitation (ChIP) assays showing the association of SIRT1, H3K9Ac, H4K16Ac, H3K27me3 and H3K4me3 to the *Tac3* promoter (**a**; *upper panels*) and *Pdyn* promoter (**b**; *lower panels*) in the medial basal hypothalamus (MBH) of female rats fed normally (NN) or subjected to postnatal nutritional manipulation (overnutrition, ON; undernutrition, UN). The results are expressed as % of the signal generated by input DNA. The bar histograms represent the mean ± SEM. *p<0.05; **p<0.01 (One way ANOVA followed by the Student-Newman-Keuls). For all panels n=4 animals/ group. NN= white bars; ON= light red bars; UN= light blue bars.

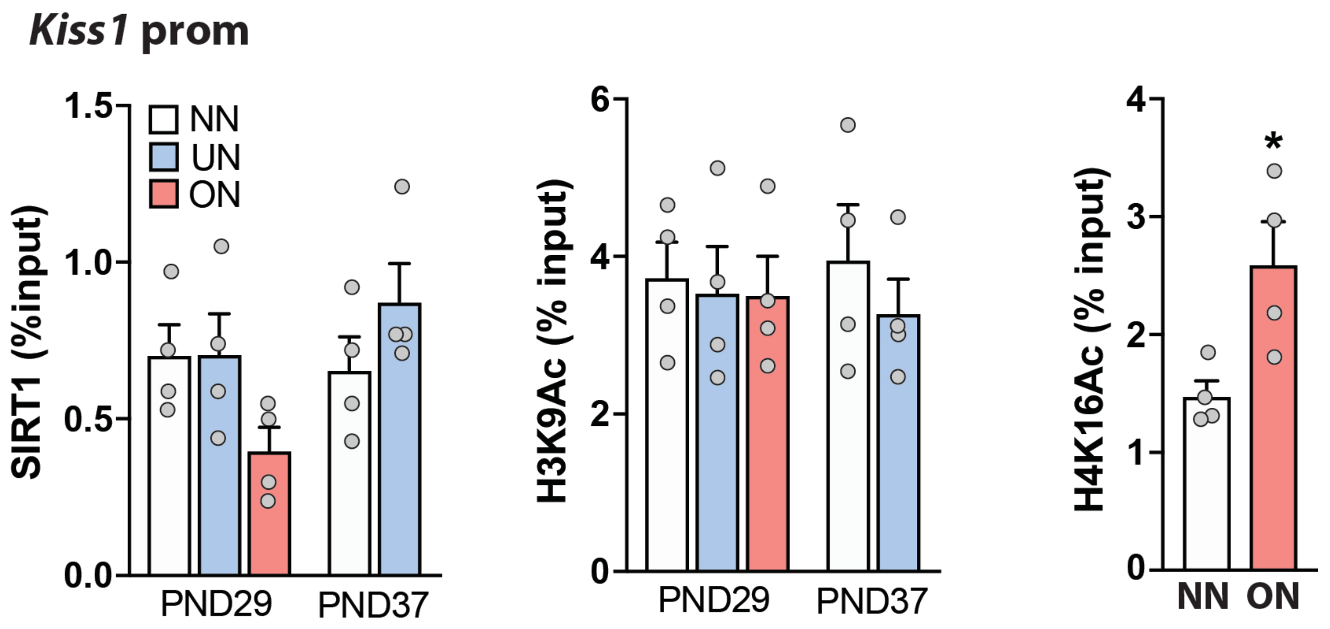

Supplementary Figure 4: Chromatin Immunoprecipitation (ChIP) assays of the association of SIRT1, H3K9Ac and H4K16Ac to the *Kiss1* promoter in the hypothalamic preoptic area (POA), encompassing the AVPV, of female rats fed normally (NN) or subjected to postnatal nutritional manipulation (overnutrition, ON; or undernutrition, UN). NN and UN samples were obtained at PND29 and PND36, while samples from ON rats were obtained only at PND29. The results are expressed as % of the signal generated by input DNA. The bar histograms represent the mean ± SEM. *p<0.05 (One way ANOVA followed by the Student-Newman-Keuls, except for right panel data: two-sided Student-t test). For all panels n=4 animals/ group. NN= white bars; ON= light red bars; UN= light blue bars.

Supplementary Figure 5: Uncropped scans of Western blots included in the study. Reference to figure and panel is include for each blot.

See the blots in the following pages.

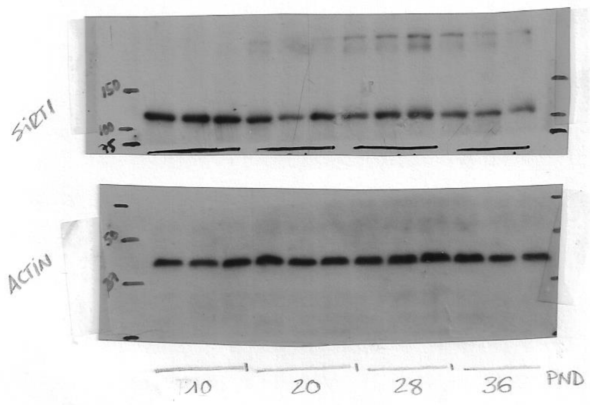

Fig 1a

WB from Figure 1.a

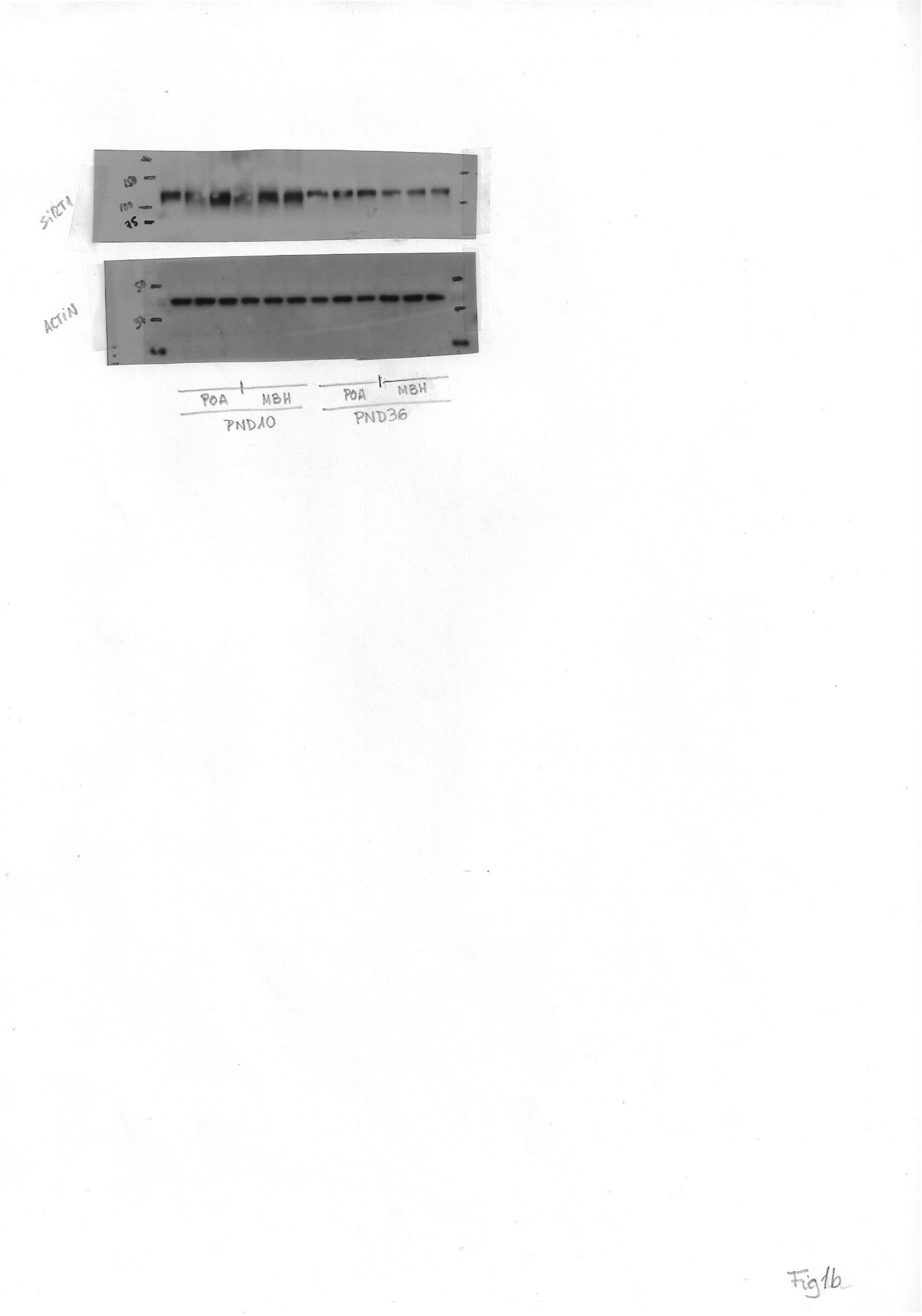

WB from Figure 1.b

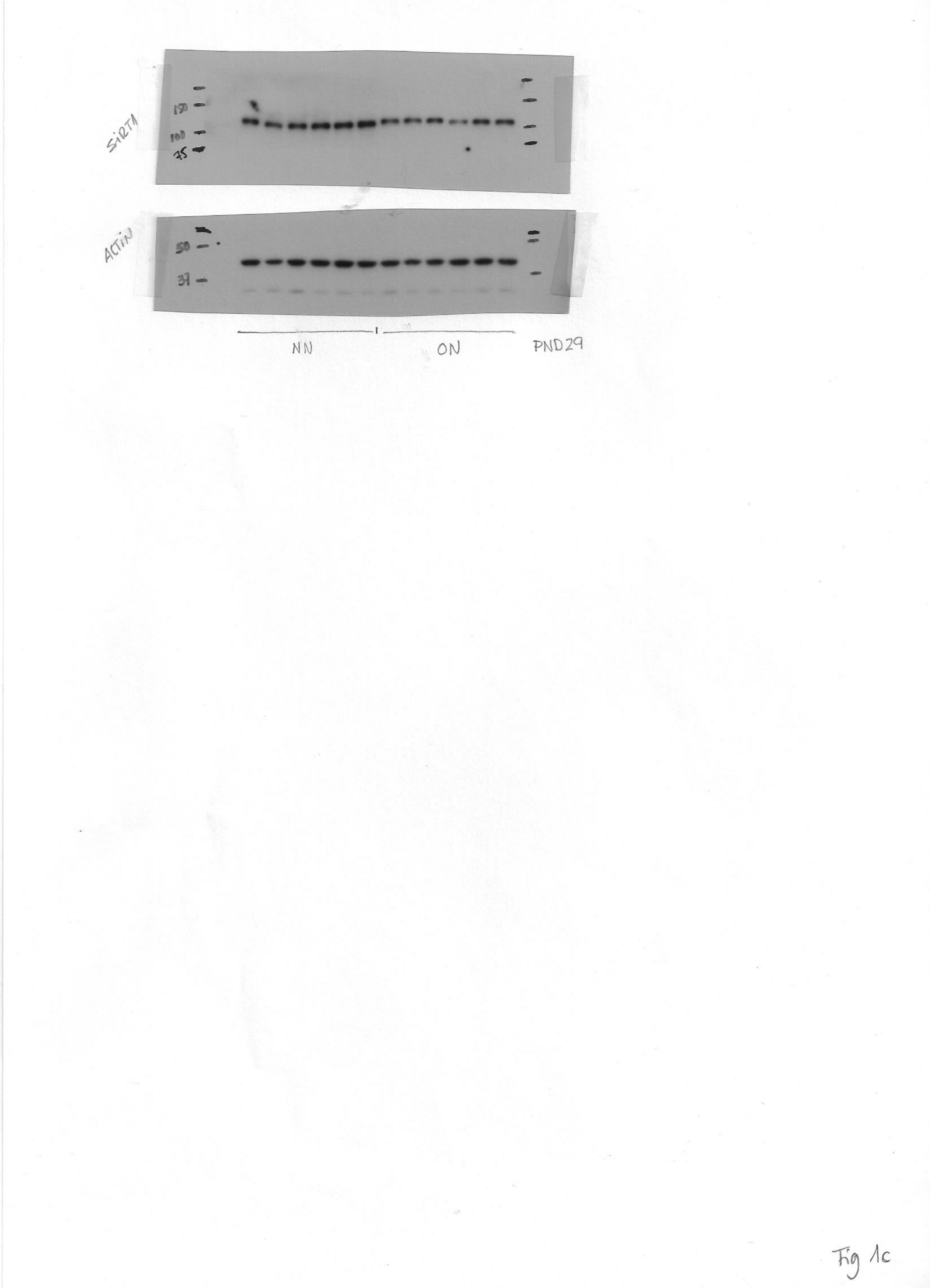

WB from Figure 1.c

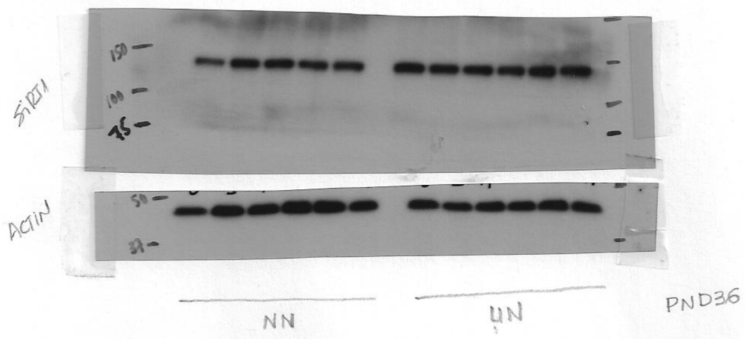

WB from Figure 2.a

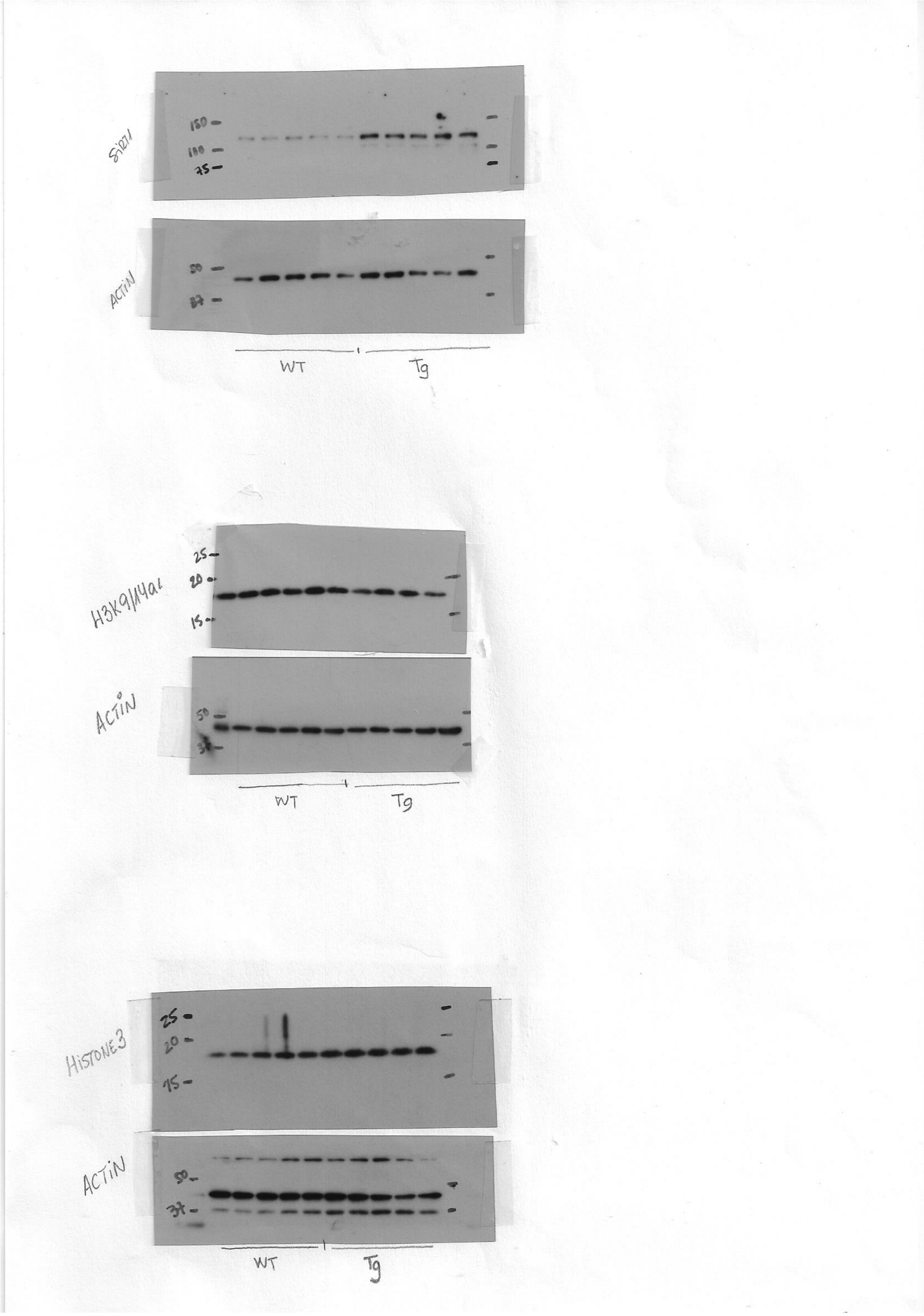

WB from Figures 3.b-c

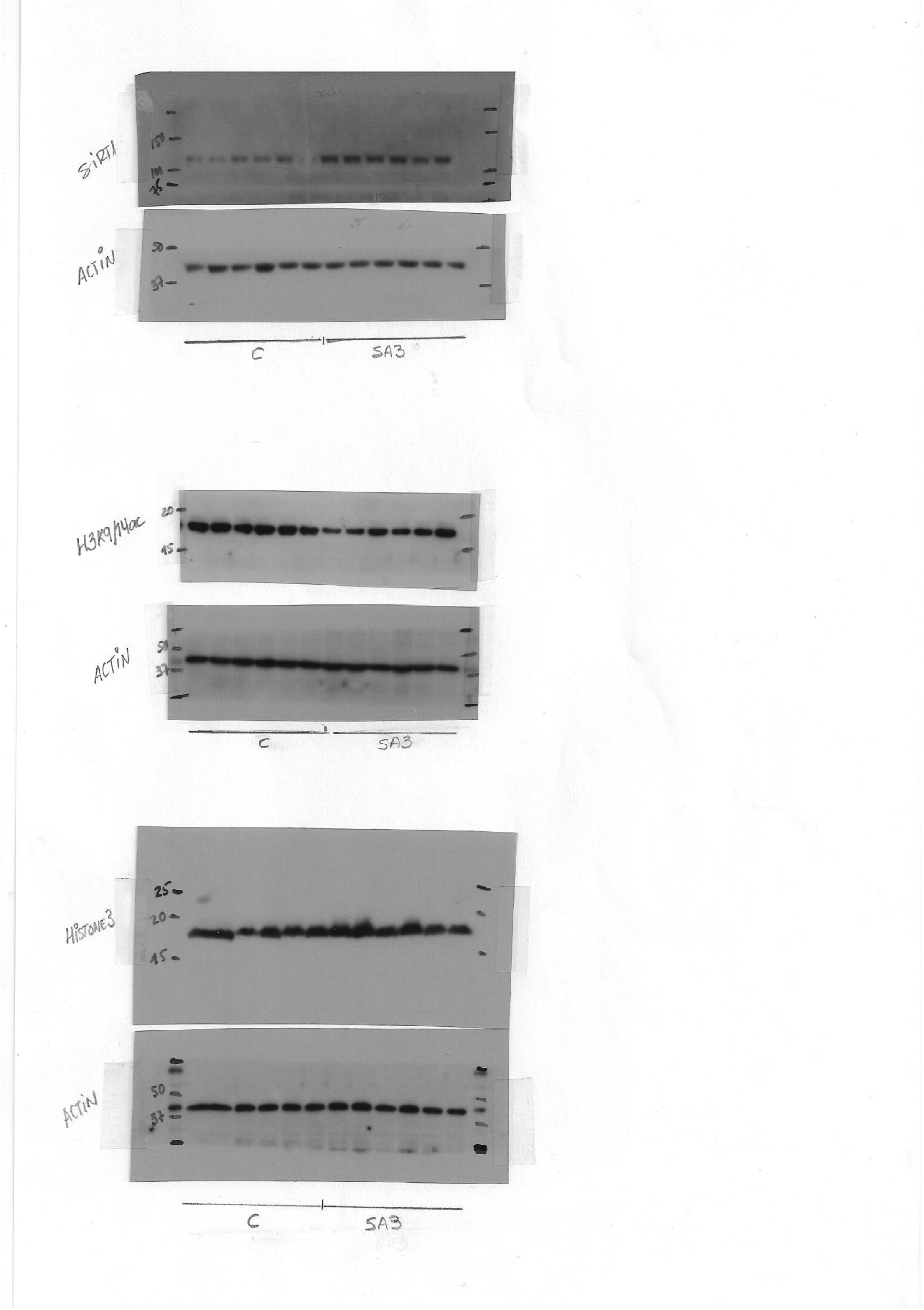

WB from Figures 4.f-g

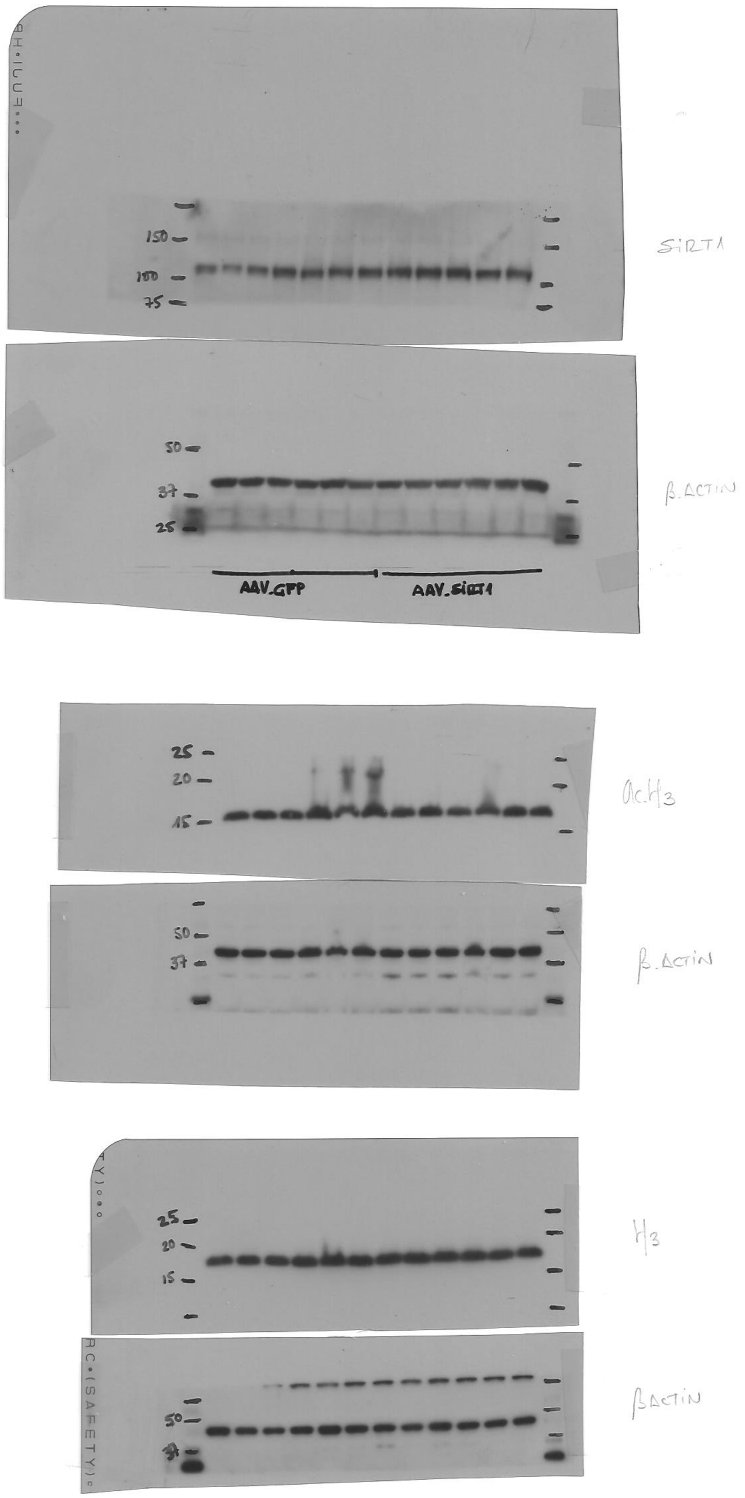

WB from Figure 5.e

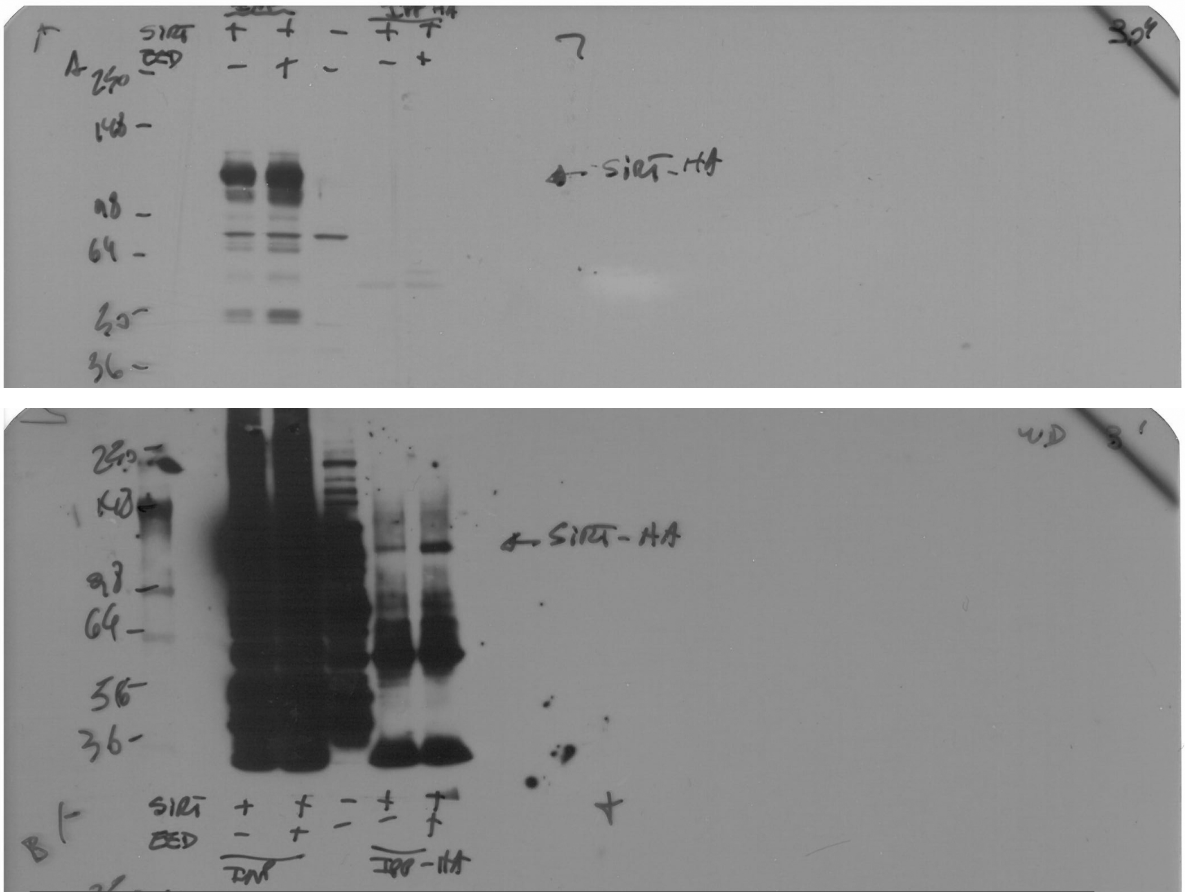

WB from Figure 8.b (left-upper panels; two different exposures of the same blot are shown)

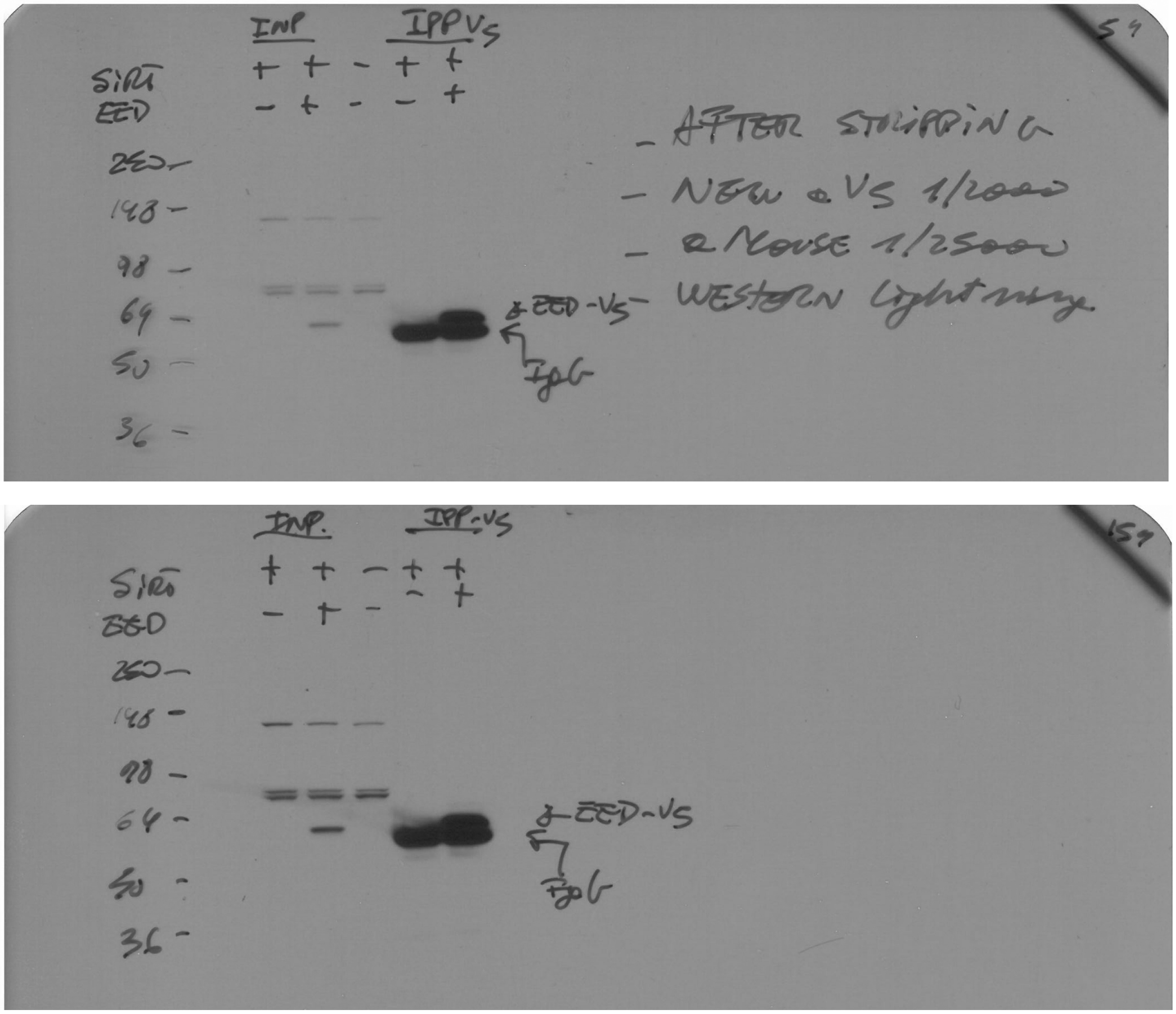

WB from Figure 8.b (left-lower panels; two different exposures of the same blot are shown)

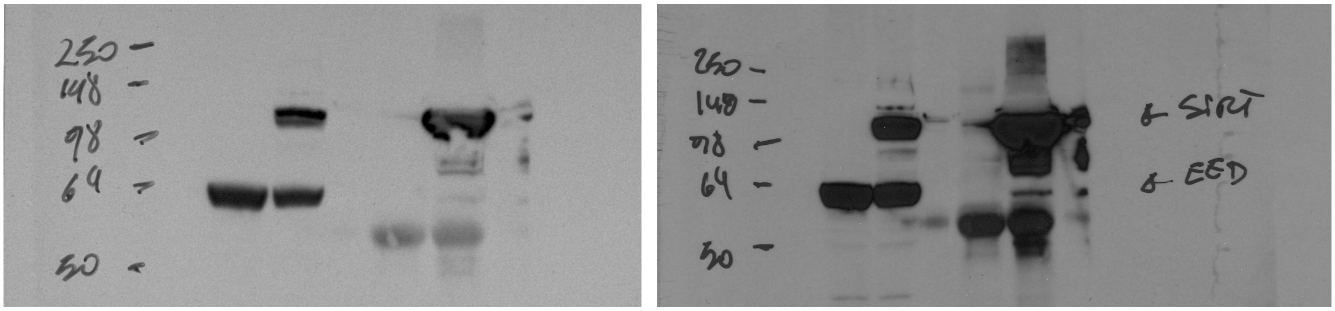

WB from Figure 8.b (right panels; two different exposures of the same blot are shown)

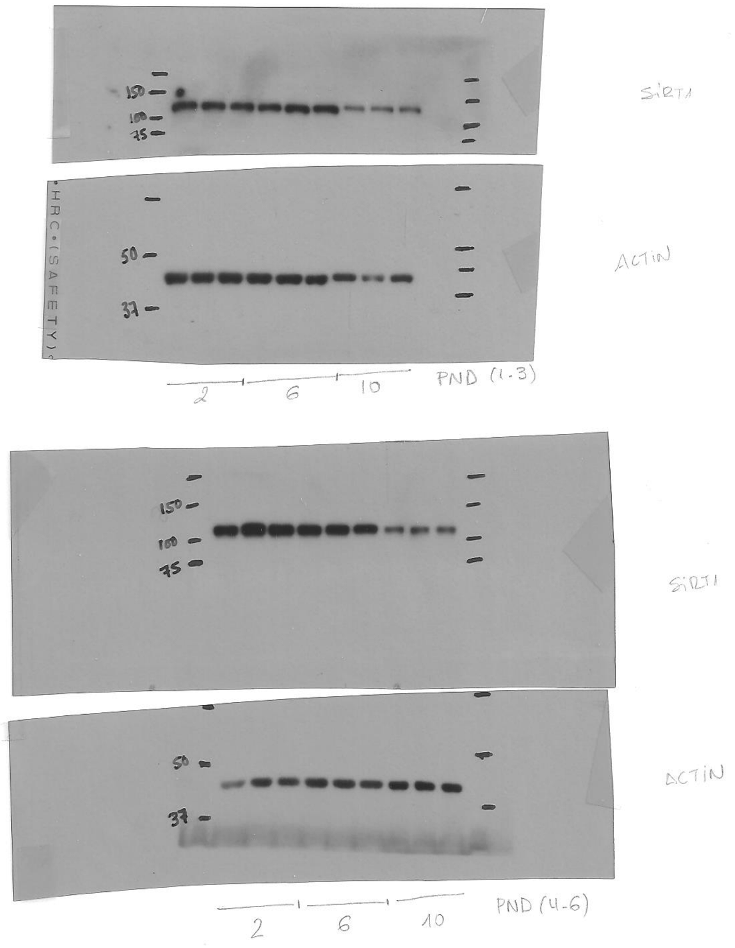

WB from Supplementary Figure 1

SUPPLEMENTARY TABLES

Supplementary Table 1: List of primary antibodies used in the different analytical methods. Source information and dilutions are also provided.

Target	Host	Source	Catalog #	Dilution/Quantity
**Chromatin Immunoprecipitation (ChIP) Assays**				
β-Galactosidase	Mouse	ICN Biomedicals	55976	3μg/reaction
β-Galactosidase	Rabbit	Cortex Biochem	CR7001RP2	3μg/reaction
EED	Mouse	Millipore	05-1320	3μg/reaction
H3K4me3	Rabbit	Active Motif	39159	3μg/reaction
H3K9ac	Rabbit	Active Motif	39917	3μg/reaction
H4K16ac	Rabbit	Active Motif	39167	3μg/reaction
H3K27me3	Rabbit	Active Motif	39155	3μg/reaction
SIRT1	Rabbit	Santa Cruz	sc-15404	3μg/reaction
**Immunohistofluorescence (IHF)**				
SIRT1	Mouse	Santa Cruz	sc-74504	1/1000
Kisspeptin	Rabbit	Millipore	AB9754	1/2000
GFP	Chicken	Abcam	ab13970	1/5000
**Immunoprecipitation (IPP)**				
HA	Rabbit	Abcam	ab9110	5μg/reaction
V5	Mouse	Thermo Fisher	MA5-15253	5μg/reaction
**Western Blot**				
SIRT1	Mouse	Cell Signaling	8469S	1/1000
Histone 3 (H3)	Rabbit	Millipore	06-755	1/5000
H3K9/14ac	Rabbit	Millipore	06-599	1/10000
β-Actin	Rabbit	Sigma Aldrich	A5060	1/5000
HA	Rabbit	Abcam	ab9110	1/1000
V5	Mouse	Thermo Fisher	MA5-15253	1/1000

Supplementary Table 2: List of primers used in the different analytical methods.

Target	Primer Name	Sequence
**Expression analyses (qPCR)**		
Rat *Kiss1*	*rKiss1-forward1*	5’-GCTGCTGCTTCTCCTCTGTG-3’
	rKiss1-reverse1	5’-GCATACCGCGGGCCCTTTT-3’
	rKiss1-forward2	5’-TGGTGAACCCTGAACCCACAGGC-3’
	rKiss1-reverse2	5’-CGGCGGGCATGGCGATGTT-3’
Rat *Tac3*	*rTac3-forward1*	5’-CAGCTTGGCATGGACCTTC-3’
	rTac3-reverse1	5’-TAGCCTTGCTCAGCACTTTCA-3’
Mouse *Sirt1*	*mSirt1-forward1*	5’-GACAGAACGTCACACGCCAG-3’
	mSirt1-reverse1	5’-TTGTTCGAGGATCGGTGCCAA-3’
Rps11 (r, m)	*Rps11-forward*	5’-CATTCAGACGGAGCGTGCTTA-3’
	Rps11-reverse	5’-TGCATCTTCATCTTCGTCAC-3’
Mouse *GAPDH*	*Gapdh-forward*	5’-CTTCTTGTGCAGTGCCAGCC-3’
	Gapdh-reverse	5’-CAAGAGAAGGCAGCCCTGGT-3’
Mouse *Ppia*	*Ppia-forward*	5’-GGCAAATGCTGGACCAAACACAA-3’
	Ppia-reverse	5’-GGTAAAATGCCCGCAAGTCAAAGA-3’
**Molecular Cloning**		
Rat *Eed*	*Eed-forward*	5’-AATAGGATCCACCAT GTCTGAGAGGGAAGTGTCGACTGCG-3’
	Eed-reverse	5’-TACTGATATCTCGAAGTCGAT CCCAACGCCAAATGCT-3’
**Chromatin Immunoprecipitation (ChIP) Assays**		
Rat *Kiss1*	*rKiss1-forward3*	5’-TCGGGCAGCCAGATAGAGGAAGC-3’
	rKiss1-reverse3	5’-TTGAGGGCCGAGGGAGAAGAG-3’
Rat *Tac3*	*rTac3-forward2*	5’-ACGTGCGTGTCTGGGTATGTGA-3’
	rTac3-reverse2	5’-GGAGGGTTTGGGGGAGTCG-3’
Rat *ProDyn*	*rProDyn-forward*	5’-CTGCCTTTCTCCTACTTTTGTCTCTGTTTT-3’
	rProDyn-reverse	5’-CGGGGGTGGATTCTCGGTGTAG-3’
**Single Cell RT-PCR**		
Mouse *Sirt1*	*mSirt1-forward2*	5’-TGACGCTGTGGCAGATTG-3’
	mSirt1-reverse2	5’-CAAGGCGAGCATAGATACCG-3’
Mouse *Kiss1*	*mKiss1-forward*	5’-TGCTGCTTCTCCTCTGT-3’
	mKiss1-reverse	5’-ACCGCGATTCCTTTTCC-3’
**In situ hybridization (Probes)**		
Mouse *Kiss1*	*rKiss1-forward4*	5’-ATGATCTCGCTGGCTTCTTGGCAG-3’
	rKiss1-reverse4	5’-TCAGCCCCGTGCCGCCCGCGC-3’
Mouse *Sirt1*	*mSirt1-forward3*	5’-ATGACAGAGCATCACACGCA-3’
	mSirt1-reverse3	5’-CTGCAACCTGCTCCAAGGTA-3’
